# Use of expert consensus to develop a shared list of procedures with potential for aerosol generation during the coronavirus disease 2019 (COVID-19) pandemic

**DOI:** 10.1017/ash.2023.118

**Published:** 2023-03-06

**Authors:** Dana E. Pepe, Preeti Mehrotra, Lou Ann Bruno-Murtha, Robert Colgrove, Shira Doron, Robert Duncan, Richard Ellison, Sarah Haessler, David C. Hooper, Michael Klompas, Cassandra M. Pierre, Thomas J. Sandora, Erica S. Shenoy, Sharon B. Wright

**Affiliations:** 1 Division of Infection Control/Hospital Epidemiology, Silverman Institute for Health Care Quality and Safety, Beth Israel Deaconess Medical Center, Boston, Massachusetts; 2 Division of Infectious Diseases, Beth Israel Deaconess Medical Center, Boston, Massachusetts; 3 Division of Infectious Diseases, Cambridge Health Alliance, Cambridge, Massachusetts; 4 Infectious Diseases Division, Mount Auburn Hospital, Cambridge, Massachusetts; 5 The Division of Geographic Medicine and Infectious Diseases, Tufts Medical Center, Boston, Massachusetts; 6 Division of Infectious Diseases, Lahey Hospital & Medical Center, Burlington, Massachusetts; 7 Division of Infectious Diseases & Immunology, UMass Memorial Medical Center, Worcester, Massachusetts; 8 Division of Infectious Diseases, Baystate Medical Center, Springfield, Massachusetts; 9 Division of Infectious Diseases; Infection Control Unit, Massachusetts General Hospital, Boston, Massachusetts; 10 Division of Infectious Diseases, Brigham and Women’s Hospital, Boston, Massachusetts; 11 Section of Infectious Diseases, Boston University Medical Center, Boston, Massachusetts; 12 Division of Infectious Diseases, Department of Pediatrics, Boston Children’s Hospital, Boston, Massachusetts; 13 Beth Israel Lahey Health, Cambridge, Massachusetts; 14 Harvard Medical School, Boston, Massachusetts; 15 Tufts University School of Medicine, Hospital, Boston, Massachusetts; 16 UMass Chan Medical School-Baystate, Worcester, Massachusetts; 17 Division of Infectious Diseases & Immunology, UMass Chan Medical School, Worcester, Massachusetts; 18 Boston University Chobanian and Avedisian School of Medicine, Boston, Massachusetts

## Abstract

The coronavirus disease 2019 (COVID-19) pandemic highlighted the lack of agreement regarding the definition of aerosol-generating procedures and potential risk to healthcare personnel. We convened a group of Massachusetts healthcare epidemiologists to develop consensus through expert opinion in an area where broader guidance was lacking at the time.

Since the beginning of the pandemic, our knowledge of severe acute respiratory syndrome coronavirus virus 2 (SARS-CoV-2), the virus that causes coronavirus disease 2019 (COVID-19), has grown considerably. At the beginning of the pandemic, SARS-CoV-2 was thought to be spread primarily through inhalation or deposition of respiratory droplets on mucous membranes,^1^ and the potential for aerosol transmission was a topic of debate. In the healthcare setting, understanding the mode of transmission is important as some procedures, often referred to as “aerosol-generating procedures” (AGPs), may increase concentrations of respiratory particles.^2,3^ This designation impacts the selection of respiratory personal protective equipment (PPE) worn by healthcare personnel (HCP), specifically whether a N95 respirator (or higher) is required instead of a medical face mask.

At the beginning of the COVID-19 pandemic, the Centers for Disease Control and Prevention based recommendations for PPE during AGPs on a review that described the transmission risk documented during the 2003–2004 severe acute respiratory syndrome (SARS) outbreak to HCP to be highest during tracheal intubation, noninvasive ventilation, tracheostomy, and manual ventilation.^4,5^ At the same time, the World Health Organization had a slightly different list of AGPs based on prior epidemiologic studies of tuberculosis and SARS,^6^ which was subsequently updated in the setting of COVID-19.^7^ Several subspecialty medical societies also developed their own guidance,^8^ with little contribution from infection prevention and control (IPC) experts. As a result, many common procedures previously not identified as generating aerosols were labeled AGPs, leading some professional societies to recommend the use of respirators.

Discussions among healthcare epidemiologists in Massachusetts at this time demonstrated agreement on the use of respirators during AGPs for patients with COVID-19. However, use of PPE varied for patients who were COVID-19 negative or of unknown COVID-19 status. In the setting of a lack of consensus definitions for AGPs, we sought to align recommendations across Massachusetts facilities by reviewing available evidence and developing guidance based on expert opinion. Our goal was to create a shared list of AGPs based on their associated risks of transmission to standardize practice.

## Methods

In total, 14 healthcare epidemiologists from 11 academic medical centers in Massachusetts convened between June and November 2020 using a consensus-based approach to develop a list of AGPs based on currently available evidence and expert opinion. This process was modeled loosely on the Delphi technique, a structured method that utilizes expert opinion to develop consensus when available knowledge is limited or uncertain.^9,10^

Prior to the first meeting, a subgroup developed a draft list of procedures for consideration based on available national and professional society guidance on AGPs, as well as inquiries from healthcare personnel that participants had received over the course of the pandemic to date. Moreover, 6 empiric categories were created to reflect different types of transmission risk: “AGPs,” “near range droplet inhalation risk,” “respiratory droplet with forced exhalation risk,” “risk of cardiac arrest requiring intubation, chest compressions or reintubation,” “unknown nonrespiratory aerosol risk,” and “not AGPs.” Each procedure on the draft list was placed into a category. In June 2020, we conducted the first email survey of the working group in 2 parts; the first assessed agreement with the categorization of each procedure and the second queried the need for an N95 respirator when performing each procedure on patients of unknown COVID-19 status or those who tested negative for COVID-19 before the procedure. A virtual meeting was held to discuss the results in early July 2020. Consensus was defined as >70% agreement.

Discussions were continued in a second virtual meeting in mid-July 2020 followed by an email survey to determine whether to use a simpler 2-category framework (ie, “AGP” vs “not AGP”) or a 3-category framework that included an “unresolved” group to capture procedures with less clear evidence of aerosol production.

A third virtual meeting was held in August 2020 to review results and determine the next steps. A final survey was conducted in September 2020 to classify “unresolved” procedures as “AGP” or “not AGP” to simplify the schema for healthcare personnel.

Responses to each of 3 surveys were shared with the group anonymously and in aggregate. Following the last survey, the final consensus AGP framework was developed and shared with partners at the MA Department of Public Health for consideration as state guidance.

## Results

**Fig. 1.**
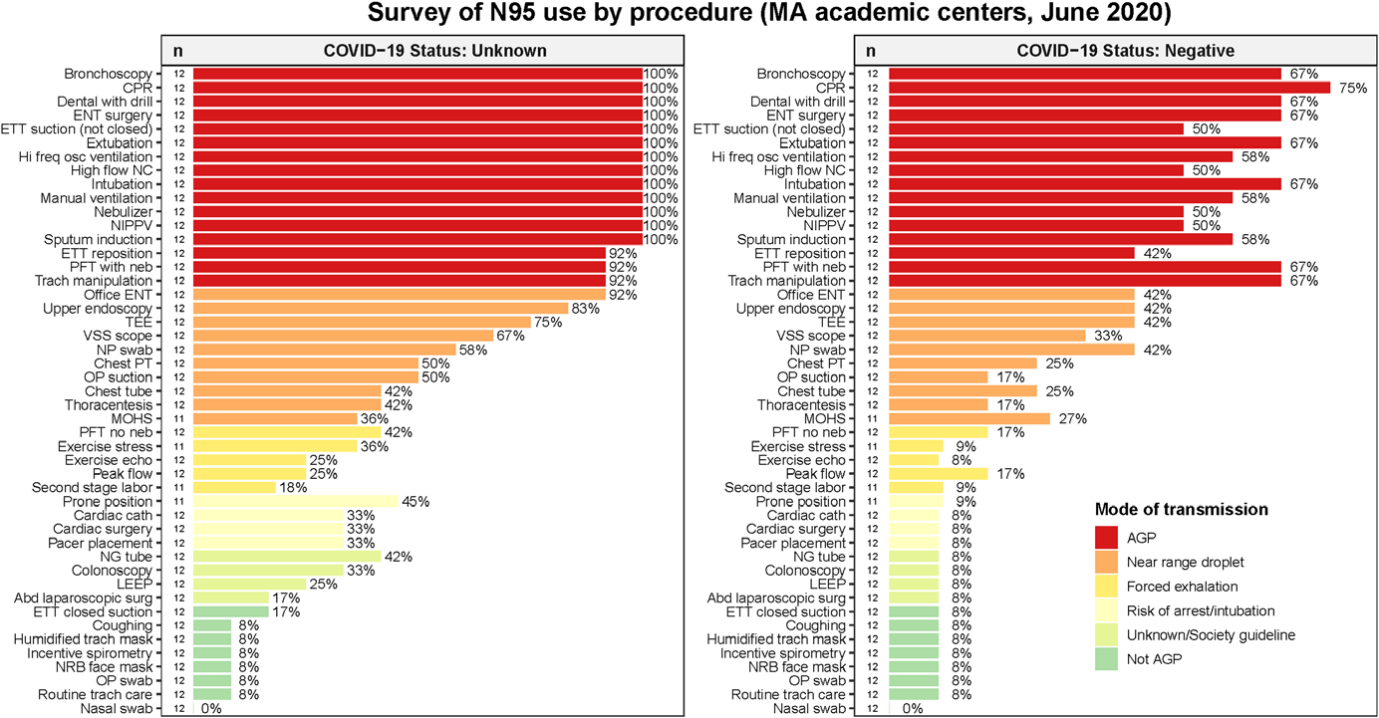
Survey of recommended N95 use, by procedure. Percentages listed in the figure represent the percent of respondents who would recommend an N95 respirator for each procedure listed for patients of either unknown or negative COVID-19 status.

In the first survey, there was high agreement on procedures that constituted AGPs and recommendation for use of N95 respirators when the patient’s COVID-19 status was unknown (Fig. 1) because they were treated similarly to patients with COVID-19. For procedures categorized as “not AGP,” the group highly agreed against recommending the use of a respirator when COVID-19 status was negative or unknown. For other procedure categories, particularly those characterized as “near range droplet,” “respiratory droplets with forced exhalation risk,” and “risk of cardiac arrest with intubation or chest compressions or need for reintubation,” the degree of agreement among the participants varied.

**Fig. 2.**
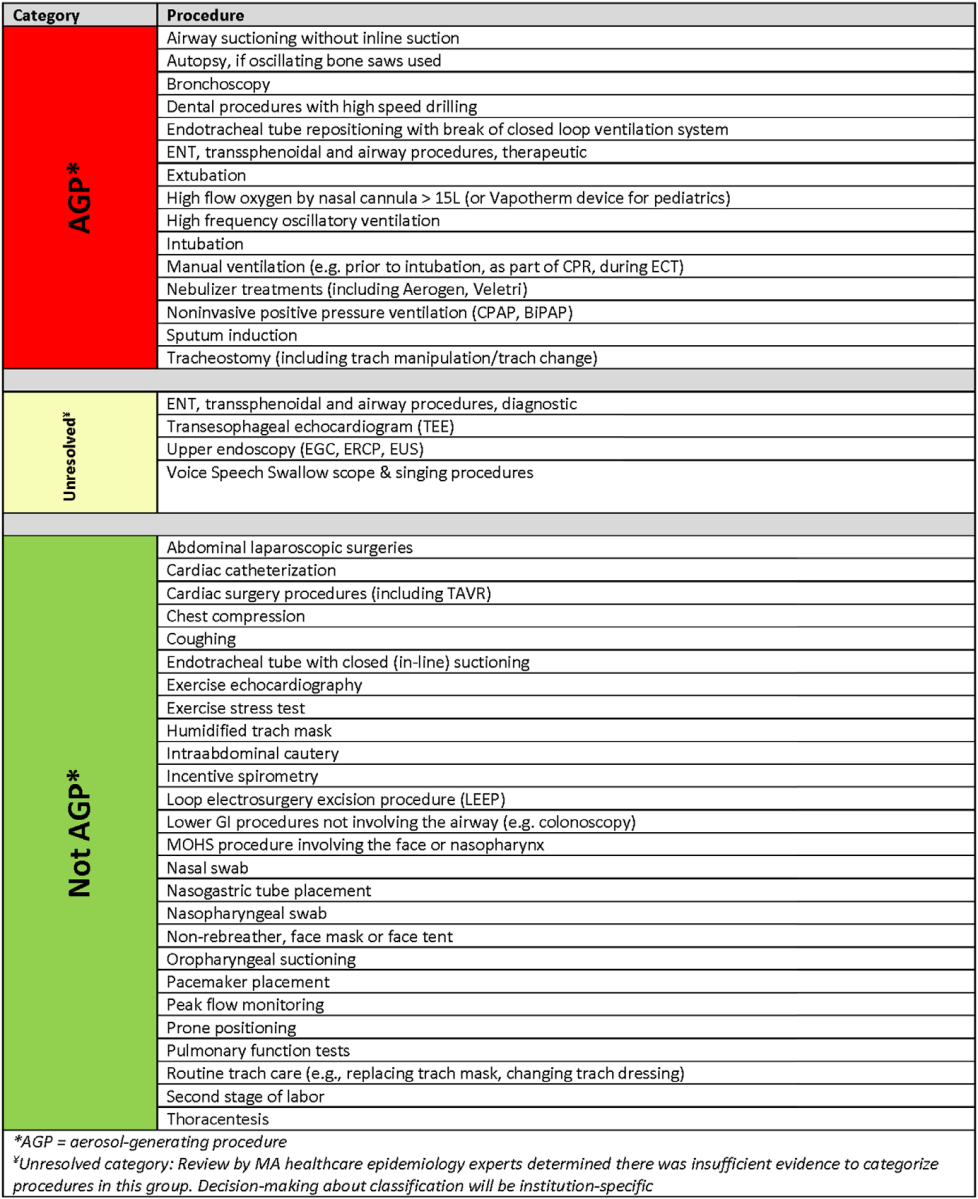
Categorization of procedures by potential for aerosol generation.

The second survey demonstrated a preference for 3 categories of procedures (8 of 14 respondents) rather than 2. The final survey focused on the 4 procedures in the “unresolved” category and demonstrated a lack of agreement to categorize these procedures as “AGP,” “not AGP” or “unresolved”: upper endoscopy (5, 6, and 3 votes, respectively); voice, speech and swallow (6, 5, and 3 votes); transesophageal echocardiogram and diagnostic ear, nose, and throat procedures (each with 7, 7, and 0 votes). Due to failure to reach >70% agreement, these 4 procedures were ultimately categorized as “unresolved” in the final framework, with the decision on respiratory protection left to each institution. Additionally, the group raised questions about 3 procedures previously classified as AGP (pulmonary function tests, high-flow nasal cannula (O_2_ ≥15L) or forced exhalation (exercise stress test)), so they were included in this vote for further clarification. The final consensus AGP framework is shown in Figure 2. Note that the list of procedures ultimately categorized as “not AGP” expanded during this process.

## Discussion

The modes of transmission of SARS-CoV-2 and the medical procedures that may carry a higher risk of potential exposure to HCP remain areas of ongoing discussion within the IPC community. We describe an approach to reach consensus through expert opinion when evidence is limited and evolving but timely decisions are necessary. The process further served as a practical means to increase awareness among healthcare institutions in Massachusetts on how others were addressing this issue. It also highlighted the important role of healthcare epidemiologists as the primary source of timely, expert advice on pathogen transmission.

Even among our group of established professionals in IPC practicing in the same state, uncertainty and differences of opinion remained about AGPs, including procedures for which consensus could not be achieved. This exercise demonstrated the value of collaboration, open-mindedness and flexibility. It also highlighted areas in which evidence was truly lacking, and in the 2 years since we completed this survey, a wealth of new data have been published about SARS-CoV-2 transmission and variable quantities of aerosol generation with different procedures. Still missing, however, are clinical data demonstrating the actual risks of infection transmission associated with various procedures. Until such data are available, this approach can be utilized to provide practical guidance when there is lack of clarity from national organizations and regulatory agencies.
